# Better Communication for Better Management of Persons with Hemophilia: Results from a Patients’-Clinicians’ Project to Address the New Therapeutic Landscape

**DOI:** 10.3390/jcm13020568

**Published:** 2024-01-19

**Authors:** Laura Banov, Silvia Linari, Luigi Ambroso, Enrico Ferri Grazzi, Samanta Gallo, Patrizio Pasqualetti, Maria Elisa Mancuso

**Affiliations:** 1 Thrombosis and Hemostasis Unit, IRCCS Istituto Giannina Gaslini, 16147 Genoa, Italy; laurabanov@gaslini.org; 2 Center for Bleeding Disorders and Coagulation, Careggi University Hospital, 50134 Florence, Italy; linaris@aou-careggi.toscana.it; 3 FedEmo—Federazione delle Associazioni Emofilici, 20151 Milan, Italy; luigi.ambroso@fedemo.it (L.A.); enrico.ferrigrazzi@fedemo.it (E.F.G.); 4 ABGEC—Associazione Bambini e Giovani con Emofilia e Altre Coagulopatie, 35128 Padua, Italy; samanta.giramondo@gmail.com; 5 Section of Medical Statistics, Department of Public Health and Infectious Disease, Sapienza Rome University, 00185 Rome, Italy; 6 Center for Thrombosis and Hemorrhagic Diseases, IRCCS Humanitas Research Hospital, 20089 Rozzano, Italy; mariaelisa.mancuso@humanitas.it; 7 Faculty of Medicine, Humanitas University, 20072 Pieve Emanuele, Italy

**Keywords:** hemophilia, hemophilia care, quality of life, Delphi method

## Abstract

Applying the Delphi method, this study aims at characterizing the perceptions and needs of physicians, individuals with hemophilia, and their caregivers in relation to the management of routine visits during regular follow-ups. A single structured questionnaire, prepared by an advisory board, was administered to 139 participants, comprising hemophilia treaters, patients and caregivers, during the period from May to June 2023. Agreement (defined following the Delphi method as developed by RAND Corporation) was reached on several topics. The Principal Component Analysis methods identified the four most relevant areas where consensus was reached among the interviewees, underscoring the necessity for in-depth discussions during routine visits: (1) medical aspects related to symptoms, life-limitations, pain, etc.; (2) non-medical related aspects (ambitions, lifestyle, network, etc.); (3) logistical-organizational aspects (home–hospital distance, alternative modalities of communication); and (4) visit duration and telemedicine integration. The results of both the Delphi and Principal Component Analysis underline that the care of individuals with hemophilia extends beyond merely prescribing drugs or treatment regimens. Instead, it necessitates consideration of numerous variables from both therapeutic and non-therapeutic domains, all of which are deemed important for the holistic management of the individuals. As a result, these aspects are routinely discussed and addressed during visits.

## 1. Introduction

The availability of innovative therapies for the management of hemophilia A (HA) has ushered in a new era, allowing continual advancements in therapeutic targets and clinical outcomes for individuals with HA (PwHA). This progress not only reduces the burden associated with the condition, but also promotes a healthier and more active lifestyle. According to the last World Federation of Hemophilia (WFH) Guidelines [1], the development of new hemostatic therapies has led to a deep revision of the concept and definition of prophylaxis. In the context of the rapidly developing hemophilia treatment landscape, assessing the impact of the condition and its treatment in a real-world context is crucial. However, this task becomes complicated if the assessment is confined to traditional outcomes. A shift from bleeding and acute outcomes to embracing a more comprehensive set of value-based outcome indicators is needed to assess the quality of care and the impact of these medical innovations [2]. This potential new approach opens the door for the development and adoption of a new clinical management model, initiating from patient–physician interaction and communication. To hold significance for the community, any significant progress in hemophilia therapies should be validated by patients, confirming that the new treatment positively impacts their daily lives and the inherently heterogenous and subjective aspect of their quality of life (QoL).

Therefore, the need to quantify and measure patient-reported outcomes (PROs) is becoming increasingly crucial.

Ongoing initiatives aim to develop patient-centric outcomes that capture meaningful changes. One specific approach, goal attainment scaling (GAS), enables patients, in collaboration with a trained clinician, to select goals from a condition-specific menu of options and subsequently facilitates quantitative assessment of goal realization [3]. Specifically, the Discrete Choice Experiment (DCE) methodology can offer stated-preference information, reveal whether particular attributes are predictors of choice in different scenarios, as well as assess the relative importance of attributes used to describe alternatives in choice sets. Evidence suggests that the DCE approach may be suitable for establishing general preferences and guiding priorities in healthcare provision [4].

This project aims to develop a practical and functional tool to facilitate discussion and interaction between PwHs and clinicians, encouraging interaction, personalized selection of topics of interest and prioritization of clinical gaps.

The project is structured in two phases:

(i) addressing and discussing topics related to the patient during the routine visit, such as QoL, pain management, psychological wellbeing, and multidisciplinary approach of patients with hemophilia by applying the Delphi methodology [5,6]. The discussants will include a heterogeneous group of hemophilia treaters, patients with hemophilia and their caregivers;

(ii) identifying the performance gaps and limits (identified by clinicians and patients during the routine outpatient visit) regarding the management of patients with hemophilia and to consequently plan a pilot project to develop a new modality to manage the physician/patient relationship via a Discrete Choice Experiment (DCE) with the ultimate aim to identify patient priorities, facilitate physician–patient discussion and improve efficiency of routine consultations.

The initial phase, as outlined in this paper, culminated in identifying the fundamental elements to form the basis for the subsequent DCE study. The attributes and levels chosen through the Delphi study will be integrated into the matrix for generating comparison tasks in a DCE. In the second phase, this DCE will undergo an initial functionality test involving 50–70 patients. The outcomes of this test, along with the evaluation of the results, will facilitate the refinement of the tool. Further fine-tuning will occur as the tool is progressively disseminated to a larger patient pool, incorporating feedback collected during this process.

The present manuscript reports the results of the first phase of the project.

## 2. Materials and Methods

### 2.1. Material Development and Study Deployment

This project employed the Delphi method, a well-established consensus-building process validated for making group-based decisions in various fields [5,7,8,9]. Traditionally based on the three core concepts of anonymity, controlled feedback, and statistical group response, the method is routinely used in health research and clinical challenges [10]. The present study took place between May and June 2023.

The project was overseen by a Scientific Board comprising 3 clinicians and 3 representatives from patient advocacy groups. They coordinated different phases of the project and contributed to the development of materials. Their tasks were as follows: (i) to analyze the scientific literature on hemophilia with particular regard to pain, QoL and follow-up; and (ii) identify the most relevant themes that are the objects of the Delphi Consensus with a panel of experts, patients and caregivers (41 physicians, 74 patients and 24 caregivers). The Delphi aimed to identify attributes for use in the DCE instrument. The Delphi consensus was administered in a single round, utilizing the Likert scale of consensus.

The survey topics were formulated and presented to a panel of responding clinicians as a single questionnaire (Supplementary Materials S1—Delphi Questionnaire). The questionnaire comprised 22 questions, designed to elicit responses on a scale ranging from 1 (maximum disagreement) to 9 (maximum agreement).

First, an advisory board of three clinical experts in this area and three patients was convened. The patients selected to join the advisory board of the project were chosen based on their direct experience with the disease and their prominent positions within their respective organizations, FedEmo (Federation of Hemophilic Associations, an organization that unites various local associations, addressing the social and clinical needs of approximately 11,000 people with hemophilia) and ABGEC (an association dedicated to raising awareness about hemophilia and other coagulopathies among children and youth).

Drawing from a comprehensive literature review, they prepared the questionnaire, which included sections on responders’ demographics.

### 2.2. Statistical Analysis

Respondents’ agreement was assessed using the Delphi method, following the guidelines established by the RAND Corporation [11]. This method requires the use of a scale ranging from 1 (maximum disagreement) to 9 (maximum agreement), with 5 corresponding to a neutral opinion about a specific item. Thus, scores given by respondents were statistically elaborated to obtain an appropriate “index of consensus”. In accordance with “The RAND/UCLA Appropriateness Method User’s Manual”, the Inter-Percentile Range Adjusted for Symmetry (IPRAS) scores, serving as a measure of score dispersion adjusted for symmetry, were utilized to determine the level of agreement for each item. The rationale is that when ratings exhibit symmetry, the Inter-Percentile Range (IPR) required to label an indication as disagreement is smaller compared to when the ratings are asymmetric. Asymmetry was defined as “the distance between the central point of the IPR and the central point of the 1–9 scale, i.e., 5”. Since the more asymmetric the ratings are, the larger the requirement of the IPR to say that there is disagreement, the following mathematical function was developed: IPRAS = IPRr + (AI × CFA), where IPRr is the IPR required for disagreement when perfect symmetry exists; AI is the Asymmetry Index; and CFA is the Correction Factor for Asymmetry. The IPRAS threshold is dependent on the symmetry of ratings about the median. Thus, each item requires a different IPRAS to be calculated. Consequently, a statement or indication is rated with a disagreement if IPRi > IPRASi. Based on IPR and IPRAS computation, it is possible to classify each statement with the appropriateness of a given diagnostic/therapeutic strategy in the following categories: Appropriate (panel median of 7–9, without disagreement), Uncertain (panel median of 4–6 or any median with disagreement), and Inappropriate (panel median of 1–3, without disagreement).

In order to determine the most relevant attributes for designing a Discrete Choice Experiment, rating scores attributed by respondents to the 22 items of the Delphi questionnaire were submitted to a factor analysis (Principal Component Analysis). The Kaiser criterion (eigenvalues of extracted factors greater than 1) was applied to obtain a data reduction. Kaiser–Meyer–Olkin Measure of Sampling Adequacy and Bartlett test for sphericity were used to assess the appropriateness of the factor analysis. While standard PCA relies on Pearson’s correlation coefficients, assuming a linear relationship between variables, it is essential to validate such assumptions. To assess potential deviations from linearity, it is necessary to explore all possible pairs of variables. In our case, involving 22 items, we should have checked 22 × 21/2 = 231 bivariate relationships. Instead of formally comparing linear and polynomial models for each of these 231 bivariate correlations, we visually compared linear relationships with those revealed by LOESS (locally estimated scatterplot smoothing). In almost all cases, no significant differences were found between the two patterns, indicating few departures from linearity.

The data analysis was conducted using SPSS 27.0 (IBM), and a scoring sheet was developed in Excel in order to calculate all the statistics as required by “The RAND/UCLA Appropriateness Method User’s Manual”.

## 3. Results

### 3.1. Study Population

The study population was composed by 139 responders, namely 41 physicians, 74 patients and 24 caregivers. The median age of the respondents was 56 years (min = 35, max = 76) for physicians, 38 years (min = 17, max = 75) for patients and 53 years (min = 19, max = 83) for caregivers. Median experience of clinicians in the management of PwHs was 20 years (min = 3, max = 40). Among the 41 physicians, 25 (61%) were hematologists, 5 (12%) pediatricians, and 6 (15%) were specialized in internal medicine. Physicians were evenly distributed across Italian regions, while patients were more commonly from Veneto.

Table 1 shows the main topics of the study and the indexes of appropriateness evaluated according to the RAND/UCLA Method.

### 3.2. Common Priorities during Visits among All Responders

Agreement was reached on several significant issues and topics that should be addressed and discussed during the routine visit (Table 1).

The first common priority was the management of everyday life issues, with special regard to:

i. non-medical related items;
ii. everyday life issues (i.e., work-related);
iii. life-style;
iv. patients’ ambitions regarding life;
v. how the distance between home and reference center may impact follow-up;
vi. alternative, non-conventional and modern communication modalities with physicians (messages, teleconsulting, videoconference, etc.) in a non-emergency setting;
vii. the achievable level of self-care and autonomy in the fulfilment of regular daily life activities and possible need of support based on age and/or joint damage;
viii. patient’s adherence to physician’s non-pharmacological indications; the potential challenges in following them and the reasons beyond these difficulties.

Agreement was also reached regarding pain management, particularly in relation to:

i. the fear of potential bleeding events;
ii. the level of acute and chronic pain with particular regard to their management;
iii. the level of joint damage with particular regard to its management, impact on daily life and on activities desired by the patients.

Responders considered several treatment-related issued to be relevant:

i. importance of comorbidities and their possible interaction with treatments potentially needed;
ii. pros and cons of different routes of administration available for hemophilia treatments;
iii. central role of the patient regarding disease management, especially related to available therapies (efficacy/tolerability, route of administration, pharmacokinetics parameters) and self-management;
iv. disease- and treatment-related limitations in every-day life activities (i.e., frequency of treatment, route of administration, logistic related to drugs transportation/conservation, etc.).

The necessity of a multidisciplinary team to address the broad spectrum of different needs among PwHs was deemed of paramount importance, with particular regard to:

i. the possibility to discuss hemophilia and its management with a multidisciplinary team;
ii. the possible psychological issues related to potential disease-related critical situations and their management within a multidisciplinary team;
iii. the possible psychological issues related to daily-life disease management or the need to deal with a disease day-by-day within a multidisciplinary team.

No consensus was reached regarding certain routine visit topics, such as:

i. its duration, regarding which both physicians and caregivers reported uncertainty on the appropriateness of the available time, with patients, on the other hand deeming it appropriate (Table 1);
ii. disease and non-disease related discussion regarding the PwH’s social networks, group supports, relationship with relatives and friends;
iii. for pediatric patients, the need/possibility of a direct dialogue between the patient and the physician without the presence of a caregiver/relative.

### 3.3. Principal Component Analysis (PCA)

The Kaiser–Meyer–Olkin Measure of Sampling Adequacy was 0.878, while the Bartlett test for sphericity yielded a value of 1900.9 (*p*-value < 0.001), indicating that PCA was applicable. The majority (95%) of off-diagonal values in the anti-image correlation matrix were below 0.2. Measures of sampling adequacy (MSA) for individual variables were assessed: the median was 0.869 (min = 0.552, max = 0.953; to be noted that the second lowest value was 0.726). The cut-off value for considering a variable relevant in explaining a factor was arbitrarily set at factor loading ≥ 0.40 (Table 2).

Through the PCA, four factors emerged as critical for discussion with the treating physician and a multidisciplinary team:

i. all the medical aspects related to symptoms, life-limitations, pain, etc.;
ii. non-medical related aspects (ambitions, lifestyle, social networks, etc.);
iii. logistic-organizational aspects (home–hospital distance, alternative modalities of communication);
iv. visit duration and telemedicine integration.

This structure was presented to the board, and 10 attributes were selected to represent each factor, describing the most plausible profiles of a care pathway.

1. Having time to talk about the most frequent and relevant symptoms: this attribute refers to the possibility of talking about all the clinical aspects that pertain to hemophilia.
2. Having time to talk about therapy management: in this case, the reference is to currently available therapies, including the one currently in use and others that could be employed.
3. Having time to talk about limitations and autonomy in daily activities: this refers to normal activities that are part of everyday life such as grocery shopping, doing housework, etc.
4. Healthcare professionals to refer to: hemophilia management requires a multidisciplinary approach, based on the expertise of several specialists. This attribute provides the possibility of consulting solely the hematologist, the hematologist and the psychologist, or a multidisciplinary team that includes, in addition to the hematologist, the physiatrist, the orthopedic surgeon, and potentially other figures who could be crucial in the clinical management of the pathology, taking into account the patient’s age.
5. Conducting visits: for a chronic condition, beyond the standard face-to-face visit, as typically conducted, a visit could be facilitated with the aid of IT tools.
6. Distance from the hemophilia treatment center (HTC): it is described based on the time required to reach the center from the patients’ home.
7. Having time to talk about social relationships, personal ambitions, lifestyle, and emotional aspects.
8. Having time to deal with co-morbidities and drug interactions: this attribute mainly refers to patients who have other diseases in addition to hemophilia and who are therefore treated with other drugs (e.g., antihypertensives, antidiabetics, etc.).
9. Having time to talk about research and pharmacological innovations: this item pertains to the discussion about new drugs in clinical trials including the possibility to be involved in clinical trials.
10. Time of the visit: the three intervals proposed are indicative of a short or unsatisfactory visit (15 min), medium or normal (30 min) and long or more thorough (45 min).

These attributes and their corresponding representative levels will be used for the design of the DCE study.

## 4. Discussion

The results of the present Delphi project can be summarized as follows:

i. Recent progress in therapeutic options led to a paradigm shift in PwH management;
ii. As condition outcomes have improved over the past decades, patients, physicians and caregivers deem everyday life-related issues as increasingly important topics to be discussed during routine visit;
iii. QoL has become a crucial priority for the PwH community;
iv. Non-disease and non-treatment related issues should be routinely discussed during follow-up visits.

The natural history of patients with severe hemophilia has profoundly changed in the last decade. New therapies have enhanced both prognosis and treatment feasibility, facilitating improved compliance and adherence [12].

Consequently, QoL has become increasingly important for PwHs, following a similar path to the recent experiences in oncology and cardiology [13,14,15,16].

This is particularly important considering that most patients with severe hemophilia are of the working age [13].

The results of the current Delphi project suggest that a broader and in-depth discussion regarding both condition-related and unrelated issues is warranted during follow-up visits. This may lead to increased disease awareness as well as improved compliance and adherence, which are crucial for achieving the main treatment goals [17].

Another relevant aspect that needs to be addressed during follow-up visits is how to improve QoL. The four elements summarized by the PCA are the main topics that have an impact on patients’ QoL: medical aspects (symptoms, life limitations, pain), non-medical aspects (ambitions, lifestyle, network, etc.), logistic aspects (home–hospital distance, alternative modalities of communication), and visit duration/telemedicine integration. Strictly related to QoL is the discussion of ambitions, expectations, and possibilities.

In this context, an important message of this Delphi project is the necessity to “normalize” the disease and streamline the patient/physician relationship. This approach aims to align the clinical management of the condition with the life plans of Persons with Hemophilia (PwH).

Therefore, in this complex scenario, a multidisciplinary team is essential to achieve optimal condition management and ensure an appropriate relationship between hemophilia treaters, PwH, and the condition itself.

The present Delphi is propaedeutic to an upcoming pilot project aimed at developing a new approach to manage the physician/PwH relationship through the Discrete Choice Experiment (DCE). This tool will aim to assist physicians and patients in optimizing follow-up visits based on patient’s priorities, allowing a tailored and personalized approach with consequent improvement of patient’s adherence and QoL.

### Limitations

Some limitations of this study should be acknowledged: (i) the overall small sample size; (ii) results cannot be generalized to broader populations; and (iii) the present project is pre-emptive for a broader project.

## 5. Conclusions

The present Delphi project showed that in a contemporary cohort of physicians, PwH and caregivers’ QoL related issues should be routinely addressed during follow-up visits within a dedicated multidisciplinary team.

If systematically integrated into routine visits, this approach would enhance a better comprehension of the perspective and needs of PwH, fostering a shared decision-making process. This should not only consider medical issues related to hemophilia and other co-morbidities, but also patients’ lifestyle, expectations, ambitions and emotional status.

If adequately analyzed, the results of this process may improve the interaction between hematologists and PwH, eventually influencing adherence to treatment and QoL.

## Figures and Tables

**Table 1:** Appropriateness Indexes evaluated according to the RAND/UCLA Method.

Question	Group	Median (Round)	IQR	IPRAS	Evaluation
1. The amount of time the doctor has available for routine visits is sufficient.	1.clinicians	5	3	2.35	uncertain
	2.patients	7	2	5.35	appropriate
	3.caregivers	6	2	3.85	uncertain
2. During the routine visit, it would be important to have time to talk about ‘other things’.	1.clinicians	8	2	6.85	appropriate
	2.patients	7	2.5	5.35	appropriate
	3.caregivers	8	3.25	6.775	appropriate
3. During the routine visit, it is important to discuss (pathology-related and non-pathology-related) issues regarding daily activities (e.g., work, everyday life).	1.clinicians	9	2	7.6	appropriate
	2.patients	8	2	6.1	appropriate
	3.caregivers	8	4	6.025	appropriate
4. During the routine hematological examination it is important to discuss (pathology-related and non-pathology-related) questions regarding one’s ambitions.	1.clinicians	8	2	6.85	appropriate
	2.patients	7	3	4.6	appropriate
	3.caregivers	8	4	4.675	appropriate
5. During the routine hematological examination it is important to discuss (pathology-related and non-pathology-related) lifestyle issues.	1.clinicians	9	1	7.6	appropriate
	2.patients	8	2	6.1	appropriate
	3.caregivers	8	2	5.35	appropriate
6. During the routine hematological examination it is important to discuss (pathology-related and non-pathology-related) issues regarding social networks, circles of acquaintances and support groups.	1.clinicians	7	2	6.1	appropriate
	2.patients	6	4	4.6	uncertain
	3.caregivers	7	3	4.6	appropriate
7. It is important that, during routine visits for pediatric patients, there is a moment for direct doctor–patient dialogue without the continuous presence of the parent/caregiver.	1.clinicians	8	2	6.85	appropriate
	2.patients	8	2.5	6.85	appropriate
	3.caregivers	7	3	4.6	appropriate
8. It is important to talk about the potential fear of bleeding events.	1.clinicians	8	1	7.6	appropriate
	2.patients	8	2	6.85	appropriate
	3.caregivers	8	3	6.85	appropriate
9. For ‘elderly’ patients, it is important to discuss clinical aspects related to advancing age (comorbidities) and possible interactions of the treatment of the condition and the condition itself with other pharmacological treatments.	1.clinicians	9	1	7.6	appropriate
	2.patients	8	1	7.6	appropriate
	3.caregivers	8	3	6.55	appropriate
10. It is important to discuss the route of administration, including the pros and cons of current treatment and potential alternative options.	1.clinicians	9	1	7.6	appropriate
	2.patients	9	1	7.6	appropriate
	3.caregivers	8	2.5	6.85	appropriate
11. The distance home–center has an impact on disease management.	1.clinicians	7	2	5.35	appropriate
	2.patients	8	4	6.4	appropriate
	3.caregivers	7	3	6.1	appropriate
12. Alternative modes of dialogue with the physician (messaging, teleconsultation, videoconferencing, etc.) may be a viable option to add flexibility, under non-emergency conditions, to the frequency of visits/contacts with the center.	1.clinicians	7	1	6.1	appropriate
	2.patients	8	2	6.85	appropriate
	3.caregivers	8	2	6.85	appropriate
13. In decisions about therapy and disease management, the role of the patient is fundamental, specifically in relation to available therapies including pharmacological characteristics (efficacy/tolerability, route of administration, pharmacokinetic parameters, etc.) and autonomy in management.	1.clinicians	8	2	6.1	appropriate
	2.patients	8	2	7.6	appropriate
	3.caregivers	8	2.5	6.85	appropriate
14. In decisions about therapy and disease management, the role of the patient is crucial, specifically in relation to satisfaction with current treatment.	1.clinicians	8	2	6.85	appropriate
	2.patients	9	2	7.6	appropriate
	3.caregivers	9	2	6.85	appropriate
15. It is important to discuss with the physician the limitations that pathology and therapy impose on everyday life (e.g., frequency, route of administration, logistics of transporting/storing/obtaining treatment, etc.).	1.clinicians	9	1	7.6	appropriate
	2.patients	8	1	7.6	appropriate
	3.caregivers	8	2	7.6	appropriate
16. It is important to be able to discuss as part of the routine visit the possibility and availability of access to a multidisciplinary team consisting of other hematologists and/or specialists with specific expertise in hemophilia.	1.clinicians	9	1	7.6	appropriate
	2.patients	8	2	7.6	appropriate
	3.caregivers	9	2	6.85	appropriate
17. It is important to discuss the level of chronic pain (or acute pain in relation to hemorrhagic events), its importance and its management.	1.clinicians	9	1	8.35	appropriate
	2.patients	9	1	7.6	appropriate
	3.caregivers	8	3	6.85	appropriate
18. It is important to discuss the level of joint damage, its management, implications for daily life and desired activities.	1.clinicians	9	1	8.125	appropriate
	2.patients	9	1	7.6	appropriate
	3.caregivers	9	1	7.6	appropriate
19. It is important to discuss during the visit the level of self-sufficiency and autonomy in performing normal daily activities and the possible need for support depending on the level of joint damage and age.	1.clinicians	9	1	7.6	appropriate
	2.patients	9	1	7.6	appropriate
	3.caregivers	8	3	6.1	appropriate
20. It is important to discuss the patient’s adherence to the doctor’s instructions (excluding drug therapy), potential difficulties in following them and the reasons for these difficulties.	1.clinicians	9	1	8.35	appropriate
	2.patients	9	2	7.6	appropriate
	3.caregivers	9	3	6.85	appropriate
21. It is important, during the routine visit, to discuss any psychological distress related to critical situations and possible management within a multidisciplinary team or with external collaborators.	1.clinicians	9	1	7.6	appropriate
	2.patients	8	2	6.85	appropriate
	3.caregivers	9	1	7.6	appropriate
22. It is important, during the routine visit, to discuss any psychological discomfort related to the day-to-day management or living with the condition and possible management within a multidisciplinary team or with external collaborators.	1.clinicians	9	1	7.6	appropriate
	2.patients	8	2	6.85	appropriate
	3.caregivers	9	1	7.6	appropriate

**Table 2:** Rotated component matrix.

Varimax Rotated Component Matrix	Factors			
	1	2	3	4
Explained variance	43.3%	11.1%	5.6%	5.1%
Items				
discuss pain management	0.863			
discuss management of joint damage	0.846			
discuss limitations from pathology and therapies	0.845			
multidisciplinary team	0.794			
modalities of treatment delivery	0.786			
discuss autonomy	0.698			
patient involvement in treatment choices	0.693			0.457
discuss adherence	0.692			
consideration of patient satisfaction with therapies	0.687			
comorbidities	0.67			
fear of bleeding events	0.645	0.458		
discuss psychological aspects of critical	0.493	0.475	0.447	
visits discuss ambitions		0.82		
visits discuss lifestyle		0.807		
visits discuss daily activities		0.702		
visits discuss social networks		0.693		
visits discuss other		0.623		
visits have doctor–pediatric–patient dialogue	0.421	0.519		
distance from home to center			0.806	
discuss psychological aspects of daily living		0.482	0.54	
time of visit				0.77
alternative modes of communication			0.456	0.514

## Data Availability

Data are available upon reasonable request.

## Supplementary Materials

The following supporting information can be downloaded at: https://www.mdpi.com/article/10.3390/jcm13020568/s1, Supplementary Materials S1: Delphi Questionnaire; Supplementary Materials S2: Contributors.
