# Supplementary material for: Better Communication for Better Management of Persons with Hemophilia: Results from a Patients’-Clinicians’ Project to Address the New Therapeutic Landscape
Source: J Clin Med. 2024 Jan 19;13(2):568. doi: 10.3390/jcm13020568 (PMC10816722; doi:10.3390/jcm13020568)
Supplement: Supplementary file 1 [file jcm-13-00568-s001.zip › Paper Better Management PwH Supplementary Material S1_Delphi Questionnaire.pdf]

Supplementary Materials S1 - DELPHI QUESTIONNAIRE

FILLING OUT THE QUESTIONNAIRE

Each question or statement should be answered by indicating your level of agreement with a score between 1 (Complete Disagreement) and 9 (Maximum Agreement).

1. **The amount of time the doctor has available for routine visits is sufficient.**
   The 'amount of time' refers to the duration of the routine consultation in which issues related to the routine management of the condition are discussed.
2. **During the routine visit, it would be important to have time to talk about 'other things'.**
   By 'other' is meant anything else that may be relevant or pertinent to the pathology, but which does not directly relate to clinical management or treatment logistics (including efficacy, convenience, adherence, etc.).
3. **During the routine visit, it is important to discuss (pathology-related and non-pathology-related) issues regarding <u>daily activities</u> (e.g. work, everyday life).**
4. **During the routine haematological examination it is important to discuss (pathology-related and non-pathology-related) questions regarding <u>one's ambitions</u>.**
   By 'ambitions' is meant all plans or aspirations concerning work activities (i.e. plans for career progression at work) or sports (plans to increase a particular activity or undertake a new one and the specific level one would like to achieve).
5. **During the routine haematological examination it is important to discuss (pathology-related and non-pathology-related) <u>lifestyle issues</u>.**
   By 'lifestyle' is meant everything related to the patient's habits (physical activity, travel, work needs [such as travel and career stage, etc.], family needs and conditions [presence of children, partners, elderly parents, etc.], etc.) and the potential implications the disease may have on these aspects.
6. **During the routine haematological examination it is important to discuss (pathology-related and non-pathology-related) issues regarding <u>social networks, circles of acquaintances and support groups</u>.**
   'Social networks' include local support groups for people/family members with haemophilia, local/national patient associations, one's own circle of friends and acquaintances with or without haemophilia.
7. **It is important that, during the routine visit, for paediatric patients there is a moment of direct doctor-patient dialogue without the continuous presence of the parent/caregiver.**
   By 'direct dialogue' we mean moments of confrontation between doctor and patient without the caregiver necessarily being present, in order to increase the patient's knowledge of the pathology and to empower him/her in the management of the pathology, thus acquiring greater autonomy.
8. **It is important to talk about the potential fear of bleeding events.**
   'Fear of bleeding events' may be related to aspects such as participation in leisure activities, sports or travel, or may be general and could potentially result in self-imposed limitations.
9. **For 'elderly' patients, it is important to discuss clinical aspects related to advancing age (comorbidities) and possible interactions of the treatment of the condition and the condition itself with other pharmacological treatments.**
   Comorbidities and pathologies specific to the elderly may be multiple (including hypertension, other cardiovascular diseases, degeneration of joint health, etc.) and may or may not be caused by haemophilia.
10. **It is important to discuss the route of administration, including the pros and cons of current treatment and potential alternative options.**
    'Route of administration' refers to how the drug is taken (e.g. intravenous, subcutaneous, intramuscular, oral, nasal) and 'alternative options' refers to alternative routes of administration for the current treatment or other available treatments.
11. **The distance home-centre has an impact on disease management.**

12. **Alternative modes of dialogue with the physician (messaging, teleconsultation, videoconferencing, etc.) may be a viable option to add flexibility, under non-emergency conditions, to the frequency of visits/contacts with the centre.**
    This statement refers to the possibility of adding to the necessary in-person visits (and thus not replacing them) an electronic contact with the physician/centre to discuss elements of disease management which do not necessarily require an in-person visit.
13. **In decisions about therapy and disease management the role of the patient is fundamental, specifically in relation to available therapies including pharmacological characteristics (efficacy/tolerability, route of administration, pharmacokinetic parameters, etc.) and autonomy in management.**
14. **In decisions about therapy and disease management, the role of the patient is crucial, specifically in relation to satisfaction with current treatment.**
15. **It is important to discuss with the physician the limitations that pathology and therapy impose on everyday life (e.g. frequency, route of administration, logistics of transporting/storing/obtaining treatment, etc.).**
16. **It is important to be able to discuss as part of the routine visit the possibility and availability of access to a multidisciplinary team consisting of other haematologists and/or specialists with specific expertise in haemophilia.**
17. **It is important to discuss the level of chronic pain (or acute pain in relation to haemorrhagic events), its importance and its management.**
    Pain management may involve pharmacological, physiotherapeutic or specialist management (consultations with pain specialists).
18. **It is important to discuss the level of joint damage, its management, implications for daily life and desired activities.**
19. **It is important to discuss during the visit the level of self-sufficiency and autonomy in performing normal daily activities and the possible need for support depending on the level of joint damage and age.**
    The 'level of self-sufficiency/autonomy' refers to the ability (or level of limitation) to carry out normal activities of daily living (e.g. cleaning, personal care, shopping, etc.) without the need for support networks (family members, professional caregivers, volunteers, etc.).
20. **It is important to discuss the patient's adherence to the doctor's instructions (excluding drug therapy), potential difficulties in following them and the reasons for these difficulties.**
21. **It is important, during the routine visit, to discuss any psychological distress related to <u>critical situations</u> and possible management within a multidisciplinary team or with external collaborators.**
    By 'critical situations' is meant any serious bleeding event, switch of therapy (for reasons of efficacy or doctor/patient choice), failure of therapy, etc.
    By 'multidisciplinary team' is meant the presence of figures dedicated to the management of these problems and the easy availability of these figures from the doctor's consultation.
22. **It is important, during the routine visit, to discuss any psychological discomfort related to the <u>day-to-day management</u> or <u>living with the condition</u> and possible management within a multidisciplinary team or with external collaborators.**
    By 'day-to-day management of the pathology' is meant any social criticism that may be caused by the pathology (e.g. stigma, embarrassment, etc.).
    By 'multidisciplinary team' is meant the presence of dedicated figures for the management of these issues and the easy availability of these figures from the doctor's consultation.
