# Supplementary material for: Better Communication for Better Management of Persons with Hemophilia: Results from a Patients’-Clinicians’ Project to Address the New Therapeutic Landscape
Source: J Clin Med. 2024 Jan 19;13(2):568. doi: 10.3390/jcm13020568 (PMC10816722; doi:10.3390/jcm13020568)
Supplement: Supplementary file 1 [file jcm-13-00568-s001.zip › Paper Better Management PwH Supplementary Material S2_Contributors.pdf]

Supplementary Materials – S2 – CONTRIBUTORS

Chiara Ambaglio, ASST Giovanni XXIII, Bergamo
Erminia Baldacci, Policlinico Umberto I, Roma
Maria Basso, Gemelli, Roma
Elisa Bertoni, ASST Spedali Civili, Brescia
Chiara Biasoli, Ospedale M. Bufalini, Cesena
Alessandra Borchiellini, AOU Città scienza e Salute, Torino
Isabella Cantori, Ospedale di Macerata, Macerata
Giuseppe Carli, ULSS 8 Berica, Vicenza
Giancarlo Castaman, AOU Careggi, Firenze
Alberto Catalano, Policlinico SS Annunziata, Chieti
Dorina Cultrera, AOU Vittorio Emanuele, Catania
Grazia Delios, SIMT ASL TO4, Ivrea
Patrizia Di Gregorio, Policlinico SS Annunziata, Chieti
Dario Di Minno, Federico II, Napoli
Paola Giordano, Ospedale pediatrico Giovanni XXIII, Bari
Anna Chiara Giuffrida, Ospedale Borgo Roma, Verona
Gaetano Giuffrida, AOU Vittorio Emanuele, Catania
Chiara Gorio, ASST Spedali Civili, Brescia
Elvira Grandone, Casa Sollievo Sofferenza, S. Giovanni Rotondo
Giuseppe Lassandro, Ospedale pediatrico Giovanni XXIII, Bari
Matteo Luciani, Ospedale Pediatrico Bambin Gesù, Roma
Renato Marino, Policlinico, Bari
Tiziano Martini, Ospedale M. Bufalini, Cesena
Angelo Claudio Molinari, IRCCS Giannina Gaslini, Genova
Mariasanta Napolitano, Policlinico Giaccone, Palermo
Paola Pedrazzi, Ospedale M. Bufalini, Cesena
Lydia Piscitelli, S. Orsola Malpighi, Bologna
Berardino Pollio, Regina Margherita, Torino
Stefania Preti, Policlinico S. Matteo, Pavia
Paolo Radossi, Ospedale S. Giacomo, Castelfranco Veneto
Vincenza Rossi, Ospedale Annunziata, Cosenza
Mariaconcetta Russo, Policlinico S. Matteo, Pavia
Cristina Santoro, Policlinico Umberto I, Roma
Rita Santoro, AO Pugliese-Ciaccio, Catanzaro
Michele Schiavulli, AORN Santobono (pediatrico), Napoli
Chiara Simion, AOU di Padova, Padova
Sergio Siragusa, Policlinico Giaccone, Palermo
Gianluca Sottilotta, Malacrino Bianchi Morelli, Reggio Calabria
Federica Valeri, AOU Città Scienza e Salute, Torino
Ezio Zanon, AOU di Padova, Padova
